# Gene Expression Changes Induced by PPAR Gamma Agonists in Animal and Human Liver

**DOI:** 10.1155/2010/325183

**Published:** 2010-10-19

**Authors:** Alexandra Rogue, Catherine Spire, Manuel Brun, Nancy Claude, André Guillouzo

**Affiliations:** ^1^UMR INSERM U991, Faculté des Sciences Pharmaceutiques et Biologiques, 35043 Rennes, France; ^2^Université de Rennes 1, 35065 Rennes, France; ^3^Biologie Servier, 45520 Gidy, France; ^4^Institut de Recherches Servier, 92150 Suresnes, France; ^5^Institut de Recherches Servier, 92400 Courbevoie, France

## Abstract

Thiazolidinediones are a class of Peroxisome Proliferator Activated Receptor *γ* (PPAR*γ*) agonists that reduce insulin resistance in type 2 diabetic patients. Although no detectable hepatic toxicity has been evidenced in animal studies during preclinical trials, these molecules have nevertheless induced hepatic adverse effects in some treated patients. The mechanism(s) of hepatotoxicity remains equivocal. Several studies have been conducted using PCR analysis and microarray technology to identify possible target genes and here we review the data obtained from various *in vivo* and *in vitro* experimental models. Although PPAR*γ* is expressed at a much lower level in liver than in adipose tissue, PPAR*γ* agonists exert various PPAR*γ*-dependent effects in liver in addition to PPAR*γ*-independent effects. Differences in effects are dependent on the choice of agonist and experimental conditions in rodent animal studies and in rodent and human liver cell cultures. These effects are much more pronounced in obese and diabetic liver. Moreover, our own recent studies have shown major interindividual variability in the response of primary human hepatocyte populations to troglitazone treatment, supporting the occurrence of hepatotoxicity in only some individuals.

## 1. Introduction

Obesity has emerged as a major health problem with 1.6 billion adults classified as overweight and obese. The condition is associated with type 2 diabetes, cardiovascular diseases, and several cancers [1] and is characterized by an increase in the size and number of adipocytes. Peroxisome proliferator-activated receptors (PPARs) act as lipid sensors and therefore represent critical molecular targets for the treatment of obesity. Thus, agonists of peroxisome proliferator-activated receptor *γ* (PPAR*γ*, also known as NR1C3) are used to treat non-insulin-dependent diabetes type 2. PPAR*γ* belongs to the superfamily of nuclear receptors; it acts as a critical transcription factor in the regulation of adipose differentiation, lipid storage, and of genes involved in energy storage and utilisation. One putative mechanism through which PPAR*γ* enhances insulin sensitivity is its ability to channel fatty acids into adipose tissue, thus decreasing plasma fatty acid concentration. PPAR*γ* can also affect insulin sensitivity by regulating hormones, cytokines, and proteins that are involved in insulin resistance [2]. It exists as two forms encoded by multiple transcript variants. PPAR*γ*1 is the predominant isoform in humans; it is highly expressed in adipose tissue but is also expressed in many other cell types in which it plays important functions, particularly intestine and immune cells. PPAR*γ*1 is the main isoform found in liver. PPAR*γ*2 is found at high levels in different adipose tissues [3]. Hepatic PPAR*γ* represents only 10–30% of the level in adipose tissue [4]. The PPAR superfamily contains two other subtypes, PPAR*α* (NR1C1) and PPAR*β*/*δ* (NR1C2). PPAR*α* is highly expressed in liver, kidney, small intestine, heart, and muscle, and it involved in fatty acid catabolism. PPAR*β*/*δ* is ubiquitous; although less studied, it is also implicated in fatty acid oxidation [5].

The mechanisms of action of PPARs have been well studied. Following activation by their ligands and heterodimerisation with retinoid X receptor (RXR), PPARs undergo specific conformational changes that release corepressors (as NcoR2/SMRT) and allow for the recruitment of coactivators (as SRC1/NCoA1, TIF2/SRC2, CBP/P300, steroid receptor coactivator 1, RIP140 (receptor interacting protein 140), PPAR*γ* co-activator-1) [6–8]. PPARs then interact with the peroxisome proliferator element (PPRE) in the promoter region of their target genes involved in lipid catabolism, fatty acid transport, and glucose homeostasis [9]. Their differential effects could be explained by the cell and promoter context as well as the availability of cofactors but also by the specific conformation changes of the receptor induced by each PPAR*γ* ligand that leads to differential promoter activation and chromatin remodelling of target genes [10].

A wide variety of natural and synthetic PPAR*γ* ligands have been identified. Besides natural ligands such as 15-deoxy-prostaglandin J2, a metabolite of prostaglandin D2 and vitamin E, PPAR*γ* agonists include several synthetic drug classes such as glitazones and tyrosine analogs. Thiazolidinediones (TZDs) are a class of PPAR*γ* agonists used in clinical practice to reduce plasma glucose level in type 2 diabetic patients. The adipose tissue is required for these agonists to exert their antidiabetic but not their lipidomic effects [11]. TZDs of the first generation were found to be highly hepatotoxic; the first one, ciglitazone (CIG), was abandoned after clinical trials and the second, troglitazone (TRO), was rapidly withdrawn from the market after reports of severe liver failure and death [12]. A second generation of PPAR*γ* agonists, rosiglitazone (ROSI) and pioglitazone (PIO), has been approved by the Food and Drug Administration (FDA) in 1999. Hepatic failures have also been observed after administration of these two TZDs but they were less frequent and severe [12]. The antidiabetic activities of another class of PPAR*γ* agonists, referred as tyrosine analogs, such as GW1929 and GW7845, looked promising but none of these compounds has been released on the market as yet [13]. 

Since dual PPAR*α* and PPAR*γ* agonists might provide broader beneficial metabolic effects through a simultaneous treatment of hyperglycemia and dyslipidemia, compounds targeting both PPAR*α* and *γ* have been developed by the pharmaceutical industry. However, the first dual agonists, muraglitazar and tesaglitazar, have been stopped during clinical trials due to cardiac and renal side-effects, respectively [14]. Other molecules are still under development, for example, drugs belonging to a new class called selective PPAR modulators (SPPARM) for the reduction of the side-effects found with glitazones, such as oedema and weight gain [15].

A major concern in the development of novel PPAR*γ* agonists that differ from the current therapeutics is their implication in tumor development in different tissues. Although, whether their activation promotes or limits this process remains unclear and may depend on specific conditions [16], the FDA requires 2-year carcinogenesis studies in rodents of new agonists prior to the commencement of clinical trials exceeding 6 months.

Major species differences exist in the sensitivity to TRO. During preclinical trials, TRO did not induce detectable hepatic toxicity in animals, including monkeys, which show similar metabolic profiles to humans [17], supporting the view that glitazone toxicity is restricted to human individuals having a particular phenotype. Consequently, it could be postulated that the use of human liver cell models represents a more suitable approach than the use of their animal counterparts for investigations of hepatotoxic effects of PPAR*γ* agonists.

Microarray technology represents a powerful tool to better understand the mechanisms of drug toxicity since it permits the identification of gene sets that are preferentially modulated after treatment. Several *in vivo* and *in vitro* studies have already been published on the effects of PPAR agonists on gene expression using different experimental conditions. However, they mainly concern PPAR*α* agonists [18–22]. Studies on PPAR*γ* agonists are limited and are usually focused on nonhepatic tissues, especially adipose tissue. We review here the effects of PPAR*γ* agonists on hepatic gene expression described in the literature using either *in vivo* animal models or *in vitro* animal and human liver cell models and make comparison with our own recent data obtained with human hepatocyte cultures.

## 2. *In Vivo * Animal Studies

### 2.1. Effects of PPAR*γ* Agonists in Normal Liver

Little information exists on gene profiling changes induced by PPAR*γ* agonists in the liver of normal animals (Table 1); this might be explained by the low expression of this receptor in this organ. Most studies relate to PPAR*α* agonist effects. However, a comparison of transcriptomic profiles of ROSI with six PPAR*α* agonists has clearly shown that this glitazone does not significantly regulate any of the PPAR*α* target genes in Sprague-Dawley rat liver [23]. In this study, Cyp4a10, a cytochrome P450 involved in lipid metabolism, was induced 14-fold by Wy-14643 (one of the most potent PPAR*α* agonists) and only 1.5-fold by ROSI. According to Memon et al. [24], the inability of TZDs to induce few PPAR*α*-responsive genes, such as the carnitine palmitoyltransferase gene (Cpt-I), suggests that they may require the presence of other coactivators or may be under dominant regulatory control of other transcription factors. However, DeLuca et al. [25] demonstrated that TZDs induce acetyl CoA oxidase (Aco) and fatty acid binding protein 1 (Fabp1), which are known as PPAR*α* target genes in both wild type and PPAR*α* null mice, without any increase of PPAR*γ* expression. In addition, it should be noted that Brun et al. [26] have observed some degree of cross-activation between PPAR*γ* and PPAR*α* with respect to the transcription of adipocyte differentiation genes, suggesting that residual PPAR*γ* receptor expressed in liver may be sufficient to mimicking PPAR*α* function.

Although preclinical animal studies have not allowed the prediction of glitazone hepatotoxicity in humans, several studies in animals have dealt with the mechanisms of TZDs hepatotoxicity. A role of Cyp 3a1 has been advanced for the enhanced acetaminophen toxicity in rats when this compound is administered with TRO [38]. However, chemical inhibition of drug metabolizing enzymes involved in TRO metabolism did not protect against TRO-induced toxicity. Another mechanism of toxicity could be the inhibition of the activity of the bile salt export pump (BSEP or ABCB11), which is responsible for cholestasis [39]. However, TRO acts largely through the induction of apoptosis and the more likely mechanism is via effects on mitochondria resulting in the depletion of ATP and the release of cytochrome c [40]. Interestingly, it has been recently shown that in a specific mouse model Sod2^+/−^ (whose mitochondrial antioxidant defense is slightly compromised), low repeated doses of TRO resulted in an oxidant injury to liver mitochondria, giving further support to mitochondria as targets for TRO-induced liver injury [41]. Nevertheless, any of the effects could explain the initiating mechanism. TZDs also have anti-inflammatory properties. They inhibit macrophage activation and down-regulate proinflammatory cytokines such as tumor necrosis factor alpha (TNF-*α*) and interleukin 6 (IL-6) in the liver of liposaccharide-stimulated mice [42, 43].

### 2.2. Effects of PPAR*γ* Agonists in Obese and Diabetic Liver

Several studies have dealt with the effects of PPAR*γ* agonists in obese and diabetic mouse liver. Enhanced levels of PPAR*γ* have been observed in the fatty liver of several animal models of obesity and diabetes, including ob/ob, db/db, A-Zip, and KKA*γ* mice [24, 29, 32]. Thus, KKA*γ* and ob/ob mice exhibit 8- and 6-fold more hepatic PPAR*γ* transcripts than C57BL/6 mice [32]. This increase was more pronounced compared to that of PPAR*α* mRNAs. On the contrary, Burant et al. [31] showed a 25% reduction in PPAR*γ* mRNA levels after TRO treatment of wild type animals. 

The mechanistic relationship between steatosis and the increase of PPAR*γ* expression in the liver is still unclear. It is possible that elevated PPAR*γ* expression in ob/ob livers appears to be a pathophysiological response to the severity state of obesity and diabetes. In this regard, transcriptional effects of TZDs on their target genes have been shown to be exacerbated in obese diabetic versus lean control animals. Indeed, some genes were overexpressed in obese and diabetic mice compared to controls after treatment with TRO; for example, adipocyte fatty acid binding protein (Ap2 or Fabp4) and fatty acid translocase (Fat or Cd36) and others were increased only in the liver of ob/ob mice, for example, the uncoupling protein 2 gene (Ucp2) [24]. The fact that PPAR*γ* target genes such as Cd36 were also induced by TRO treatment in lean mice without induction of PPAR*γ* expression [24] suggests that glitazone effects in obese or diabetic rodent models are different from those occurring in lean control animals. Therefore, it is essential to estimate glitazone effects in regard to the metabolic status of the animals. Noteworthy, the genes found to be modulated in the liver after glitazone treatment were unchanged in the adipose tissue [24]. 

Some differences have been observed among glitazone effects. Thus, only ROSI was found to induce an increase of both liver weight and hepatic triglycerides in AZIP/F1 mice. This could be explained by PPAR*γ*-independent mechanism effects [11]. However, another explanation is the higher affinity of ROSI than TRO for the PPAR*γ* receptor. Indeed, ROSI caused higher incidence and severity of microvesicular steatosis in obese KKA*γ* mice compared to TRO due to its higher receptor affinity (approximately 100-fold) and its higher transcriptional response. In that study, the hepatic triglycerides content of treated and untreated animals was not different, leading to the conclusion that this microvesicular steatosis is not due to triglyceride accumulation. Exacerbation of fatty liver has also been reported with ROSI which exerted its effects on serum glucose levels independently of hepatic PPAR*γ* levels [44]. Compared to wild type ob/ob mice, triglyceride content as well as mRNA levels of lipogenic genes, such as fatty acid synthase (Fasn), acetyl CoA carboxylase (Acc), and stearoyl CoA desaturase 1 (Scd1), were strongly decreased in corresponding PPAR*γ*-deficient animals [44]. These data indicate that obese mice are more sensitive to the steatogenic effects of glitazones than lean animals.

It has been shown that a few PPAR*α* target genes, such as Aco which is involved in peroxisomal *β*-oxidation, were deregulated in diabetic rodent models after TZDs treatment. Since PPAR*α* and PPAR*γ* recognize similar DNA response elements, it is quite conceivable that TZDs could modulate PPAR*α* responsive genes in liver of obese mice [45]. In wild type rodents, TRO and ROSI cause a decrease in serum cholesterol, triglycerides, free fatty acid content and, obviously, glucose levels without modifying liver or body weight [11, 23, 31]. Their effects on these biological parameters are substantially higher in obese mice. 

To understand the effects of increased PPAR*γ* expression in fatty liver cells, Yu et al. used PPAR*α* ^−/−^ mice and explored gene effects of PPAR*γ*1 overexpression [27, 28]. Expression of genes involved in adipocyte differentiation and lipid metabolism was modulated in the liver of this KO mouse model. Noticeable increase was observed in Cd36, glucokinase (Gk), malic enzyme (Me), low-density lipoprotein (Ldl), microsomal transfer protein (Mtp), and angiopoietin-like 4 (Angptl4) in the absence of any change in CCAAT/enhancer binding protein alpha (C/ebp*α*), sterol regulatory element binding protein 1 (Srebp1), phosphoenolpyruvate carboxykinase (Pepck), and glucose transporter type 2 (Glut-2 or Slc2a2) expression levels, leading to the conclusion that there is an adipogenic conversion of the liver when PPAR*γ*1 is overexpressed in this organ. Moreover, Vidal-Puig and coworkers demonstrated that forced expression of PPAR*γ*2 or *γ*1 in fibroblasts was sufficient to drive the determination of an adipocyte cellular lineage [46]. Furthermore, the relative abundance of PPAR*α* in normal liver might serve as a key regulator of fatty acid catabolism, thereby minimizing the need for pathological adipogenic transformation of hepatocytes to store lipids. PPAR*α* and fatty acid oxidation activity might partially protect from too high PPAR*γ*1 adipogenic activity in the liver. Way et al. analyzed transcript levels of genes involved in lipid and glucose homeostasis in Zucker Diabetic Fatty (ZDF) rats and concluded that PPAR*γ* activation had coordinate effects on genes involved in important hepatic metabolic pathways such as Pepck and glucose 6 phosphatase (G6P) which were decreased [30].

### 2.3. Extrahepatic Effects of PPAR*γ* Agonists

Marked tissue-differences are observed in the response to glitazones: relative to the liver and the skeletal muscle, PPAR*γ* is 10- to 30-fold higher expressed in human and rodent adipose tissues [47]. Likewise, while PPAR*γ* agonists affect only a small number of genes in the liver and the skeletal muscle, they cause conspicuous changes in gene expression in adipose tissues [30]. Thus, following a 14-day treatment of ZDF rats, ROSI decreased Tnf-*α* and increased glucose transporter 4 (Glut4), muscle carnitine palmitoyl-transferase (Cat), stearoyl CoA desaturase (SCoA), and fatty acid translocase (Fat) in adipose tissue, while only Fat was slightly augmented in the liver which expresses very little hepatic PPAR*γ*. Comparison with the effects of retinoid X receptor-selective agonists, such as LG100268, that also produce insulin sensitization in diabetic rats, showed that these agonists modulated different gene patterns from those observed with ROSI, indicating that these compounds may act by independent and tissue-specific mechanisms [48]. 

Similar tissue-differences were observed in diabetic (db/db) mice treated with PIO for 2 weeks. Analysis of 42 genes associated with diabetes by RT q-PCR showed that in the liver, expression of Gk, Glut-2, apolipoprotein A-IV (ApoA-IV), PPAR*γ*, and a series of fatty acid oxidation enzymes were increased while those of triglyceride lipase, lipoprotein lipase, apolipoprotein A-I (Apo-AI), and insulin receptor substrate 2 (Irs-2) were decreased [49]. 

Glitazones decrease glucose concentrations not only by their action on adipocytes but also by their effects on the liver and muscle. Indeed in aP2/DTA mice, whose white and brown fat is virtually eliminated by fat-specific expression of diphterin toxin A chain, TRO alleviated hyperglycemia without affecting PPAR*γ* levels in liver, suggesting independence from both adipose tissue and PPAR*γ* receptor [31]. However, conflicting observations have been reported. Thus, after mouse treatment with PIO, gene expression of Pepck was found to be increased in the liver by Hofmann et al. [50] but only in muscle by Suzuki et al. [49]. Accordingly, increased expression of PPAR*γ* in the liver of diabetic mice has been reported in certain studies [46]. 

PPAR*γ*1 or *γ*2 mRNA levels are not affected in adipose tissue by obesity in the ob/ob and Gold ThioGlucose (GTG) animal models. Accordingly, Auboeuf et al. [51] demonstrated that obesity and non-insulin-dependent diabetes mellitus are not associated with alteration in PPAR*γ* gene expression in adipose tissue in humans. However, conflicting observations have been made. Indeed, Vidal-Puig et al. [46] showed that expression of PPAR*γ*2 mRNA is increased in adipose tissue of obese men and women, and that the ratio of PPAR*γ*2/*γ*1 is directly correlated with their body mass index. In addition, they did not observe similar changes in muscle. 

Besides its well-known function in adipocyte differentiation, PPAR*γ* activation by TZDs leads to an anti-inflammatory response in adipose tissue. This has been observed in fat deposits of various obese or diabetic rodent models [52] and in fat biopsies of type 2 diabetic patients [53]. This anti-inflammatory response can be assessed by the inhibition of expression and/or biological activity of several proinflammatory factors such as TNF*α*, IL-6, plasminogen activator inhibitor 1 (PAI-1), monocyte chemoattractant protein 1 (MCP-1), and angiotensinogen [54]. Proposed molecular mechanisms, underlying this effect, include inhibition of the intracellular NF-kappaB pathway [55] and activation of nuclear translocation of the glucocorticoid receptor [56]. 

Macrophages accumulate in adipose tissue of obese animals, where they can produce inflammatory mediators, contributing by this way to insulin resistance [57]. Targeted deletion of PPAR*γ* in macrophages severely impaired TZDs response in mice submitted to a high fat diet [58]. These data emphasize the crucial role of macrophages for obtaining full effects of TZDs in the context of insulin resistance or in diabetic conditions.

Despite its weak expression level, PPAR*γ* is thought to play a role as a regulator of insulin action in the skeletal muscle [59]. Indeed, it has recently been shown that muscle-specific PPAR*γ* deletion in mouse caused insulin resistance [60]. PIO treatment of a murine model of myoblasts, the C2C12 cells, improved insulin sensitivity as assessed by increased glucose uptake [61]. Moreover, some data indicate that PPAR*γ* activation in skeletal muscle could contribute to the beneficial effect of TZDs. Indeed, experiments on myocyte models have shown that ROSI induced local expression of the insulin-sensitizing hormone adiponectin [62]. However, conflicting results on the role of PPAR*γ* in muscle have also been published, showing that muscle specific PPAR*γ* KO did not impair TZD action in a mouse model of insulin resistance [63]. These results raise the question of which tissues are really necessary to achieve pharmacological action of TZDs. A precise analysis of PPAR*γ*  regulation in other tissues where its expression reaches a sufficient level could lead to an answer.

## 3. *In Vitro * Animal Studies

Cytotoxicity studies have shown that primary rat hepatocytes were not more sensitive to TZDs than cells that did not express the drug metabolizing enzymes involved in their metabolism. TRO was more toxic than ROSI and PIO at equimolar concentrations [12]. TRO induced a decline in mitochondrial transmembrane potential and apoptosis as well as an oxidative stress. These effects were also observed in other cell types and on isolated mitochondria [64].

Several studies have been carried out on modulation of gene expression by TZDs in rodent hepatocytes (Table 1). Different concentrations and exposure times have been tested although a 24h-treatment was the most frequent. Using Applied Biosystem rat genome survey microarrays with 26857 probes, Guo et al. [12] compared the effects of five PPAR*γ* agonists, including TRO, CIG, ROSI, and PIO, in rat hepatocytes after a 6-hour treatment. Around 2-fold more genes were modulated in TRO- and CIG- than in ROSI- and PIO-treated cell samples. Genes related to cell death were deregulated only with the most cytotoxic TRO and CIG concentrations. Similar observations were reported by Vansant et al. [36]. TRO was also found to modulate more genes than other glitazones (ROSI, PIO) at the same concentration, especially genes related to oxidative stress, DNA repair, and cell death, such as heme oxygenase 1 (Ho-1), NAD(P)H quinone oxidoreductase (Nqo), growth arrest DNA-damage-inducible 45 (Gadd45), FBJ osteosarcoma oncogene (Fos), BCL2-like 11 (Bcl2l11), and BH3 interacting domain 3 (Bid3). A TRO response closer to CIG than to the second TZDs generation, ROSI and PIO, was also found in the C9 rat liver cell line [36].

As observed *in vivo* [31], TRO induced expression of the PPAR*γ* gene [33] and repressed genes related to lipid metabolism, such as Fasn and Cebp/*α*, in cultured rat hepatocytes [34]. Cyp induction by TZDs was evidenced in cultured primary rat hepatocytes using RTq-PCR analyses. Thus Cyp 3a and 2b subfamily genes were increased after exposure to TRO, ROSI and PIO [35, 36, 65]. Other genes including multidrug resistance (Mdr) 2 and 3, cadherin and superoxide dismutase (Sod) 2, were also up-regulated while Mdr1 and organic anion transporting polypeptide 8 (Oatp 8) were down-regulated [34]. 

PPAR*γ*2 expression was shown to induce lipid accumulation in the mouse AML12 liver cell line stably expressing PPAR*γ*2, and several genes known to be overexpressed in steatotic liver of ob/ob mice were found to be up-regulated by TRO, such as adipose differentiation-related protein (Adrp), Fabp4, Srebp1, Fasn, and Acc by q-PCR analysis. Lipid accumulation and the lipid droplet protein were further increased after a 7-day treatment with TRO [37]. 

An extensive study of gene expression changes induced by TZDs has also been performed on the mouse 3T3-L1 adipocyte cells using microarrays and RTq-PCR [9]. Expression gene profiles obtained with TRO, ROSI and PIO tested at concentrations that elicited maximum biological effects (i.e., 20 *μ*M for PIO and TRO and 1 *μ*M for ROSI) were distinct but with an overlapping: 94 out of the 326 deregulated genes were found to be modulated by the three glitazones after a 24-hour treatment. For example, pepck, pyruvate dehydrogenase kinase 4 (Pdk4) and c-Cbl-associated protein (Cap) were activated by the three compounds but with different time-curves, suggesting different mechanisms of gene regulation. Moreover, ROSI and PIO were more potent than TRO in activating Pepck and Pdk4 and repressing regulator of G-protein signaling 2 (Rgs2). These data support the conclusion that gene profile changes induced by TZDs are different in liver cells and in adipocytes, in agreement with *in vivo* observations.

## 4. *In Vitro * Human Liver Cell Studies

Most studies on the effects of TZDs in human liver have been performed with primary hepatocyte cultures or hepatoma cell lines. Primary human hepatocytes are recognized as the most pertinent *in vitro* model but they exhibit early phenotypic alterations and their survival does not exceed a few days in standard culture conditions. Human hepatocytes have, in addition, a scarce and unpredictable availability and are characterized by large interdonor functional variability [66]. Hepatic cell lines were thought to be an alternative but most of them have lost most of, if not all, their bioactivation capacity and consequently are of limited interest. In this regard, the new human hepatoma HepaRG cell line seems as an exception [67]. HepaRG cells exhibit a capacity of transdifferentiation; they undergo morphological and functional features of liver bipotent progenitor cells after plating and at subconfluence, lose expression of progenitor markers and differentiate into either hepatocyte-like or cholangiocyte-like cells [68]. Differentiated HepaRG cells possess most functional activities of primary mature hepatocytes and the indefinite growth capacity of hepatoma cells [69]. Noteworthy, they express major cytochromes P450, conjugating enzymes, and plasma transporters [70]. 

The mechanism(s) of TZD hepatotoxicity in humans still remain(s) controversial. Several proposals have been advanced to explain the induction of apoptosis by TRO namely accumulation of toxic metabolites or bile acids, mitochondrial damage, and oxidative stress. TRO has been shown to be metabolized by CYP3A4 (the homolog of Cyp3a1 in rodent) to a very active quinone metabolite which is able to produce reactive oxygen species via the redox/cycling or to bind to cellular proteins [40]. This CYP3A4-mediated metabolism is in accordance with the frequent occurrence of centrilobular necrosis of the liver. CYP3A4 is also induced by TRO in primary human hepatocytes [71, 72] and a correlation has been observed between CYP3A4 levels and hepatocyte sensitivity to glitazone [73].

Studies dealing with the effects of TZDs on gene expression in either primary human hepatocyte cultures or human hepatoma cell lines are scarce and most of them studied only few genes [76, 79] (Table 2). Human hepatocytes were slightly more sensitive than their rat counterparts [34], but less sensitive than human hepatoma HepG2 cells to cytotoxicity induced by TRO, supporting the conclusion of an absence of correlation between TRO toxicity and its hepatic metabolism. TRO was found to induce cell arrest and to cause time- and concentration-dependent apoptosis in various liver cell types [75, 77, 83]. Cell arrest was associated with increased expression of a cascade of cyclin-dependent kinase inhibitors, that is, cdki p21, p27, and p18 that each plays a crucial role in adipocyte differentiation through PPAR*γ* activation [81]. This increase occurred through down-regulation of nuclear S-phase kinase-associated protein 2 (SKP2) [80]. Apoptosis was associated with activation of both c-Jun N-terminal protein kinase and p38 kinase and overexpression of proapoptotic proteins and cyclooxygenase 2 (COX-2) [77, 83]. These effects are not limited to hepatoma cell lines. Indeed, TRO also induced growth arrest of prostate and bladder carcinoma cell lines [84]. By contrast, other members of the glitazone family, ROSI or PIO, had no effect on the growth of these cell lines [84] and did not cause any apoptosis of HepG2 cells [83]. The endogenous ligand 15-deoxy-prostaglandin J2 was also found to inhibit growth of prostate and bladder carcinoma cell lines by inducing apoptosis [84]. Since these effects are selective of the PPAR*γ* ligand and the cell line, they can be interpreted as PPAR*γ*-independent effects [84]. Apoptosis induced by TRO in human MCF-7 breast carcinoma cells has been associated with induction of GADD45 gene expression [85] while growth inhibition has been correlated to overexpression of another DNA damage gene, GADD153, in nonsmall lung carcinoma cells [86]. 

As observed in rat hepatocytes, Kier and coworkers [87] showed that TRO induced more genes than ROSI in human hepatocytes. This observation was based on analysis of gene expression profiles and did not include individual characterization of deregulated genes. Other studies have shown a down-regulation of SREBP-2, a gene encoding the sterol regulatory element-binding protein-2 that mediates cholesterol synthesis, as well as the two SREBP-2 target genes, 3-hydroxy-3-methylglutaryl-Coenzyme A reductase (HMGCR) and low density lipoprotein receptor (LDLR), in HepG2 cells exposed to 30 *μ*M TRO for 4 h [78]. In agreement with *in vivo* human data, TZDs were also found to modulate CYP activities in human hepatocyte cultures. CYP3A4 and CYP2B6 were induced by TRO [34] and only CYP2B6 by ROSI and PIO [35] in primary human hepatocytes. Similar observations were made in the well-differentiated HepaRG cell line [74].

**Figure 1:**
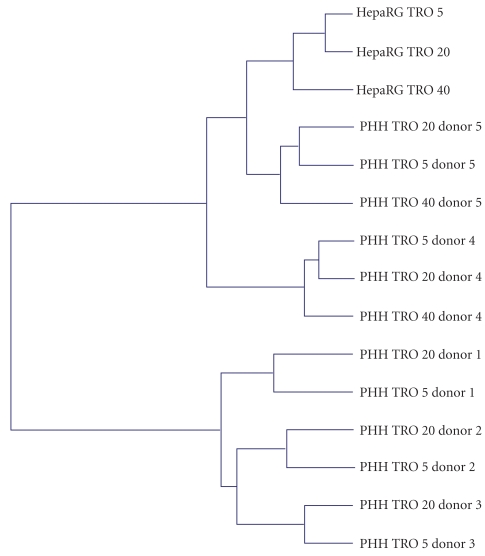
Two-dimensional hierarchical clustering of gene expression profiles induced by TRO treatment in primary human hepatocytes from five donors and HepaRG cells. The clustering was generated by using the Resolver system software with an agglomerative algorithm, the Ward's min variance link heuristic criteria, and the Euclidean distance metric. Cultures and microarray analysis as in Table 2.

**Figure 2:**
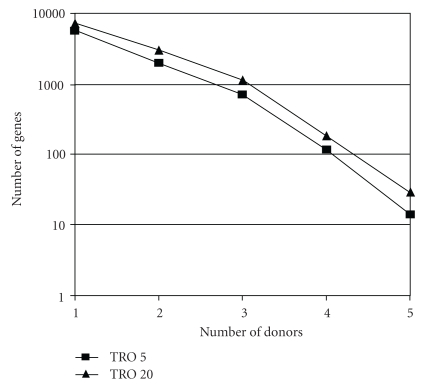
Total gene numbers modulated by 5 and 20 *μ*M TRO in primary human hepatocytes from one to five donors (FC ≥ 1.5 pv ≤ 0.01). Cultures and microarray analysis as in Table 2.

Up to now, studies on human hepatocytes have been limited to a few donors (one to three), and no interdonor variability has been considered. Since interdonor variability in response to chemical inducers or inhibitors is well established, we recently compared the effects of TRO in human hepatocyte cultures from five donors after a 24-hour treatment using pangenomic microarrays (Rogue et al., unpublished data). Two-dimensional hierarchical clustering of gene expression profiles showed that hepatocyte populations separated according to the donor and not to the TRO concentration (Figure 1). It exhibited two separate clusters: one with donors 4 and 5 and the second with donors 1, 2, and 3. The number of genes modulated by TRO greatly varied as a function of the donor and drug concentration. At 5 and 20 *μ*M, TRO modulated 5754 and 7266 genes, respectively, in at least one donor but only 4 and 29 genes in the 5 donors, respectively (Figure 2). The small subset of common deregulated genes in hepatocyte cultures from several donors is in agreement with the findings reported by Goyak et al. [88], showing that the number of modulated genes deregulated in ten populations of human hepatocytes by arochlor 1254, di(2-ethylhexyl)phthalate, and phenobarbital did not exceed 0.1%. In our study, among the few genes deregulated in the five donors by 5 *μ*M TRO, only two genes involved in oxidative stress, namely, mannose binding lectin 2 (MBL2) and serum/glucocorticoid regulated kinase 2 (SGK2), were induced. Genes involved in lipid metabolism, such as FABP1 were deregulated only by 20 *μ*M TRO in all the donors. Several PPAR target genes, such as CYP4A1, CPT1, or CD36, were induced in the two hepatocyte cultures treated by 40 *μ*M TRO.

Despite its therapeutic indications, TRO only slightly affected transcription of genes involved in glucose homeostasis. Fructose-1,6-bisphosphatase 1 (FBP1), an enzyme involved in gluconeogenesis, was not modulated in any of the five donors, in agreement with previous observations [34] while PDK4 and PEPCK were differently regulated across the donors. Their transcription could be either induced, repressed, or not affected by TRO treatment.

Comparison of gene profiles after TRO treatment in five human hepatocyte populations and the well-differentiated human hepatoma HepaRG cells evidenced a clear separation between the two cell models by two-dimensional hierarchical clustering. HepaRG cell samples separated as a function of TRO concentration and the dendrogram showed that they were closer to donors 4 and 5 than to donors 1, 2, and 3. The number of commonly modulated genes between HepaRG cells and primary human hepatocytes increased with the drug concentration; it was higher than the number of commonly modulated genes in four out of five donors. Among them, genes involved in lipid metabolism, such as FABP4 or CD36, were induced. Taken altogether our data support the view that the effects induced by TRO and more generally by PPAR*γ* agonists are quite variable from one individual to another. This could explain the occurrence of toxic effects in only few treated patients. However, whether it would be possible to predict their potential hepatotoxicity in some patients on the basis of analysis of the expression level of a peculiar gene subset requires further studies.

## 5. Conclusions

Despite the numerous published studies on TZDs, their pharmacological and toxicological effects still remain obscure. Adipose tissue seems to be a predominant target organ. However, achievement of TZD pharmacological efficiency is obtained not only through an adipose-mediated mechanism but also requires an action in other organs, notably liver and skeletal muscle and also, as recently reported, in macrophages [58]. Moreover, PPAR*γ*  itself is required in a majority of metabolic tissues for regulation of insulin action and normal physiologic response to nutrients and it plays a critical role in the development of steatosis.

The determinants of susceptibility to glitazone-induced idiosyncratic hepatotoxicity remain to be elucidated. Studies have mainly concerned the most cytotoxic compound TRO. Evidence has been provided that toxicity is not directly related to its metabolism and the generation of a quinone metabolite. Direct toxicity caused by mitochondrial dysfunction has been demonstrated using both hepatic and nonhepatic *in vitro* models. Whether hepatotoxic effects of TZDs are related to PPAR*γ* activation is not clear. PPAR*γ* is only poorly expressed in the liver and both dependent and independent effects of glitazones have been seen. 

Most *in vitro* studies have been performed with TRO concentrations of 50 *μ*M or more while maximum plasma concentrations reached 3 to 6 *μ*M in humans, making questionable the extrapolation of *in vitro* data to the *in vivo* situation. In comparison to TRO, ROSI is less toxic. The daily dose necessary for TRO therapeutic efficacy was 200 to 600 mg/day while it is only 4 to 8 mg/day for ROSI, indicating that patients were exposed to quite different doses between the first and second generations of TZDs [89].

During the last years, some studies have been designed to identify potential hepatic target genes in *in vivo* and *in vitro* models using RT-qPCR and microarray technologies. The amount of available data is still limited and has been obtained from different experimental conditions. However, some genes have been found to be usually modulated, being mainly related to drug and lipid metabolism. Interestingly, we have observed massive interindividual variability in the response of primary human hepatocytes to TRO treatment that could reflect the human situation. However, much more work is needed in order to identify the more pertinent genes which are differently expressed in the human hepatocyte populations and to determine whether the most critical effects in the liver are dependent or not on PPAR*γ* activation. Moreover, it would be important to estimate the effects resulting from long-term repeated glitazone treatments and to determine if intracellular PPAR*γ* levels in human liver cells are a critical parameter. The possible long-term treatments of differentiated normal and steatotic human hepatoma HepaRG cells could represent a unique way to better understand hepatotoxicity of PPAR*γ* agonists.

## Figures and Tables

**Table 1:** A literature survey of gene expression changes after treatment of *in vivo* and *in vitro* rodent liver models with PPAR*γ* agonists.

		*IN VIVO*														*IN VITRO*						
	Ref.	[27]	[28]	[29]			[24]	[30]	[11]		[31]		[23]	[32]		[33]	[34]	[12]	[35]	[36]	[36]	[37]
	model	PPAR*α* ^−/−^ mice PPAR*γ*1 overexpression		AZIP	WT LKO	AZIP LKO	ob/ob	ZDF rats	WT mice	AZIP	WT	ap2/dta	sprague dawley rats	KKA	ob/ob	primary rat hepatocytes					c9 rat	aml-12 murine
	treatment	TRO 5 d 0.1% v/v	no treatment	ROSI 5 w 5 mg/kg/d			TRO 10 d 200 mg/ 100 g chow	GW 1929 7 d 5 mg/kg/d	ROSI 3 w 3 mg/kg/d		TRO 5 w 16.6 mg/g chow		ROSI 4 d 30 mg/ kg/d	ROSI 28 d 2.5 mg/kg/d and TRO 100 mg/kg/d d		TRO 6 h 10– 200 *μ*M	TRO 24 h 5–50– 200 *μ*M	TRO, ROSI, PIO, CIG 6 h–10 h 5–200 *μ*M	TRO 24 h 25 *μ*M	TRO, ROSI, PIO 24 h 10–50– 100 *μ*M	TRO, ROSI, PIO, CIG 6 h–10 h 5–200 *μ*M	TRO 5 d 5 *μ*M
	method	affymetrix/q-PCR		northern blotting				northern blotting/ q-PCR			northern blotting			q-PCR		northern blotting	q-PCR	applied biosystem	q-PCR	q-PCR/eXpress		q-PCR
	Por																			+		
Oxidative stress	Hmox1																	+		+	+	
	Ephx2						−															
	Nqo																			+	+	
	Acox1			+	−	+																
	Cox2																				+	
	Serpine1	−																				

	Kif5b	+																				
Cell cycle, proliferation and death	Brca1	+																				
	Cdkn1c	+																				
	Gadd45a	−																				
	Kif24	+																				
	Gadd153																	+			+	
	Atf3																	+				
	Bcl2l11																	+				
	Blnk																	+				
	p21	+																			+	
	Gadd45g																	+		+	+	
	Cflar																	+				
	Egr1																	+				
	Emp2																	+				
	Fem1b																	+				
	Fhl2																	+				
	Fos																	+				
	Herpud1																	+				
	Hmga1																	+				
	Igfbp1																	+				
	Igfbp6																	+				
	Il7																	+				
	Lats1																	+				
	Mt1a																	+				
	Nr1d1																	+				
	Phlda1																	+				
	Sfn																	+				
	Skil																	+				
	Stk17b																	+				
	Bid3																	+				
	Ckn1b																	+				
	Cldnk4																	+				
	Myd116																	+				
	Trp53inp1																	+				
	S100a10		+																			
	Kif5b	+																				
	Il7	−																				
	Actn1																+					
	Cdh1																−					
	Gsn																−					
	Ucp2	+					+															
	Fsp27	+	+																			
Lipid metabolism	Acaca			+	−	+																
	Lipe		+																			
	Srebp1							0	0													+
	Fabp4	+	+				+			+												+
	Adfp			+	−	+																
	Cd36	+	+	+	−	+	+															
	Cpt1a			+	−	−	0		0	0												
	Cpt1b			+	−	−	0		0	0												
	Pex11a	+																				
	Acsl5							+														
	Apoc3						0															
	Dci							−														
	Elovl4	+																				
	Fabp5	+																				
	Fasn			+	−	+		+		−												+
	Ldlr			+	+	−																
	Lipc			+	+	+																
	Acdl							−														
	Thiolase		+					−														
	Kiaa1881	+	+																			
	Lamb3		+																			
	Me1							+					+									
	Mttp			−	−	−																
	Peci							−														
	Pnpla2	+																				
	Scd1			+	−	+				+												

	Cyp1a1																		0			
Xenobiotic metabolism	Cyp1a2	−																	0			
	Cyp2b1																		+	+		
	Cyp2b2																		+			
	Cyp2b6																					+
	Cyp3a																		+			
	Cyp3a1																			+		+
	Cyp3a3																+					
	Cyp4b1		+																			
	Cyp4a10	+	+										+									
	Cyp4a14	+	+																			
	Cyp51a1							+														
	Gsta2	−	−																			

	G6pc							−														
Carbohydrate metabolism	Pdk4	+																				
	Pepck							−														
	Gk							+														
	Irs2								−													
	Pc	+	+					−														
	Aqp7	+																				
	Abcb1		+																			
Transporters	Abcc3:																+					
	Slco1b1	+																				
	Slco1b3																−					
	Mdr2																+					
	Slco4a1	+																				
	Slc25a1							+														

	Nr1i3																+					
Nuclear receptors	Hnf4a																−					−
	Ppara			+	−	+			0	0												
	Pparg		+	+	−	+				+	−	+				+		+				
	Cebpa																−					
	Pparg1													+	+							
	Pparg2														+							
	Ces3		−																			
	Hspa1																	+				
	Il6r																	+				
	Vnn1	+																				
	Scd2																+					
	Actg1							+														
	Adipoq	+	+																			
	Cav1	+	+																			
	Cav2	+																				
	Cfd	+	+																			

	Lrp1			+	+	+																
Miscellanous	Mapk		+																			
	Paqr7	+																				
	Tmem159	+																				
	Tpm2	+																				
	Aco						+															
	Aox			+	+	+			+	+												
	growth response protein												+									
	Fdft1							+														
	Hex												+									
	Hmgcs2							−														
	Igh-6							+														
	Il8bp		−																			
	Ldh-a												+									

+: up-regulated

−: down-regulated

0: not modulated

The case is empty when the gene has not been studied.

**Table 2:** Modulation of gene expression in human liver cell models after treatment with PPAR*γ* agonists.

	Ref.	[34]	[35]	[65]	Rogue et al. unpublished															[74]	[75]	[76]	[77]	[78]	[79]	[80]	[81]
	model	PHH			PHH DONOR 1		PHH DONOR 2		PHH DONOR 3		PHH DONOR 4			PHH DONOR 5			HepaRG cells			HepaRG cells	Hep3b Huh7	Huh7	Hepg2			HLF	HLF, HAK, HuH-7
	treatment	TRO 24 h 5,50,100 *μ*M-	TRO 24 h 25 *μ*M-	TRO 24 h 10 *μ*M-	TRO 24 h 5 *μ*M-	TRO 24 h 20 *μ*M-	TRO 24 h 5 *μ*M-	TRO 24 h 20 *μ*M-	TRO 24 h 5 *μ*M-	TRO 24 h 20 *μ*M-	TRO 24 h 5 *μ*M-	TRO 24 h 20 *μ*M-	TRO 24 h 40 *μ*M-	TRO 24 h 5 *μ*M-	TRO 24 h 20 *μ*M-	TRO 24 h 40 *μ*M-	TRO 24 h 5 *μ*M-	TRO 24 h 20 *μ*M-	TRO 24 h 40 *μ*M-	TRO 24 h 0,024 *μ*M–25 *μ*M	TRO04 8 h - 50 *μ*M-	TRO-ROSI 1–8– 24 h 50 *μ*M-	TRO 6 h 30 *μ*M-	TRO-4 h 10–30 *μ* M	TRO-ROSI 48 h 25–100 *μ*M-	TRO for up 48 h 50 *μ*M	TRO 24 h 50 *μ*M
	method	q-PCR		Amer-sham	Agilent												Agilent			q-PCR		Super Array Bioscience qPCR	q-PCR/ northern blotting	q-PCR			
	ABCB1	+			0	+	0	0	+	+	0	0	+	+	+	+	0	0	0								
Transporters	ABCC2	+			0	0	0	0	0	0	0	0	0	0	0	+	0	0	0								
	ABCC3	+			+	0	0	0	0	0	0	0	0	0	0	0	0	0	0								
	SLC10A1	−			0	0	0	0	0	0	0	0	0	0	0	−	−	−	−								
	ABCB4	+			0	0	0	0	+	+	0	+	+	+	+	+	0	0	0								
	SLCO1B1	0			0	0	0	0	0	0	0	0	0	0	0	0	0	0	−								
	SLCO1B3	−			−	−	−	−	−	−	0	−	−	0	−	−	0	0	0								

	CCND1	+			0	+	+	+	0	+	0	0	0	0	0	0	0	0	0								
Cell cycle, proliferation, death and differentiation	CDKN1A	0			0	0	+	+	+	+	0	0	0	0	0	0	0	0	0							+	+
	GADD45G	+			0	+	0	0	0	+	0	0	0	+	+	+	0	0	0								
	AFP	−			0	−	0	−	0	0	0	−	−	0	0	0	0	0	0								
	TGFA	+			0	0	0	−	−	−	0	0	0	0	0	0	0	0	0								
	CCNE1				0	0	0	0	0	0	0	0	0	0	0	0	0	0	+							0	0
	ALB	0			0	0	0	0	0	0	0	0	0	0	0	0	0	0	−								
	CDKN1B				0	0	0	0	0	0	0	0	0	0	0	0	0	0	0							0	0
	JUND	0			0	0	0	0	0	0	0	0	0	0	0	0	0	0	0								
	CCNG1	0			0	0	−	−	0	−	0	0	0	0	0	0	0	0	0								
	MYC	0			0	0	0	0	0	0	0	0	0	0	0	0	0	0	0								
	TGFB1	0			0	0	+	+	0	+	0	0	0	0	0	0	0	0	0								
	ALPL	0			0	0	0	0	0	0	0	0	+	0	0	0	0	0	−								
	GADD45A	+			0	0	0	0	0	0	0	0	0	0	0	0	0	0	0								
	IGFBP1				0	0	0	−	−	−	0	0	0	0	+	0	0	0	+						+		
	SKP2				0	0	0	0	0	0	0	0	0	0	0	0	0	0	+							−	

	FABP1	+			+	+	+	+	+	+	0	+	+	+	+	+	+	0	−								
Lipid metabolism	FASN	+			0	0	0	0	0	0	0	0	0	+	+	0	0	0	0								
	CPT1A	+			0	+	+	+	0	0	0	+	+	+	+	+	0	0	0								
	SREBF2				+	0	0	+	0	0	0	0	0	0	0	0	0	0	0					−			
	HMGCR				0	0	0	0	0	0	0	0	0	0	0	0	0	0	−					−			
	INSIG1				0	0	0	0	0	0	0	0	0	+	+	+	0	0	0					nd			
	LDLR				0	0	0	0	0	0	0	0	0	0	0	0	0	0	0					−			
	INSIG2				0	0	0	0	0	−	0	0	0	0	0	0	0	0	0					+			

	CYP1A1		0		+	+	0	0	0	+	0	0	0	0	0	0	0	−	0	0							
Xenobiotic metabolism	CYP1A2	+	0		0	0	−	0	0	0	0	0	+	0	0	+	0	0	0	0							
	CYP2B6			+	+	+	0	0	+	+	0	+	+	0	0	+	0	0	0	+							
	CYP2C9	+			0	0	0	0	0	0	0	0	+	+	+	+	+	0	0								
	CYP2E1	+			0	0	−	−	−	−	0	0	0	0	0	0	0	0	−								
	CYP3A4	+	+		0	0	0	0	+	+	0	+	+	+	+	+	+	+	+	+							
	UGT1A10	+			0	0	0	0	0	0	0	0	0	0	0	0	0	0	0								
	GSTP1	+			0	0	0	0	−	−	0	0	0	0	0	0	0	0	0								
	GSTA1	0			0	0	0	0	0	0	0	0	0	0	0	0	0	0	−								
	G6PC				0	0	0	0	−	−	0	0	0	0	0	0	0	−	−								
Carbohydrate metabolism	PDK4				−	0	−	−	−	−	0	+	+	+	+	+	0	+	+								
	PEPCK				0	0	0	0	−	−	0	0	0	0	0	+	+	+	+								
	FBP1	0			0	0	0	0	0	0	0	0	0	0	0	0	0	0	−								

	HMOX1	+			+	+	+	+	+	+	+	+	+	0	0	0	0	+	+								
Oxidative stres	PTGS2				0	0	0	0	0	0	0	0	0	0	0	0	0	0	0		−		−				
	HSPA1A	+			+	+	0	0	+	+	0	0	0	0	0	0	0	0	0								
	TXN	+			0	0	0	0	0	0	0	0	0	0	0	0	0	0	0								
	COX-2																				−						
	CAT				−	0	0	−	−	0	0	0	0	0	0	0	0	0	0			+					

	HNF4A	+			+	0	0	0	0	0	0	0	0	0	0	0	0	0	0								
Transcription factors	PPARG				0	0	0	0	0	0	0	0	0	0	0	0	0	0	0		+			0			
	CEBPA	0			0	+	+	+	+	+	0	0	0	0	0	0	0	0	0								
	CEBPB	0			0	+	0	0	0	0	0	0	0	0	0	0	0	0	0								
	NR1I2	0			0	0	0	0	0	0	0	0	0	0	0	0	0	0	0								
	NR1I3	0			0	0	0	−	0	0	0	0	0	0	0	0	0	0	−								

	GSN	0			0	0	0	0	0	0	0	0	0	0	0	0	0	0	0								
Fbrosis/ senescence	TIMP1	0			0	0	0	0	0	0	0	0	0	0	0	0	0	0	0								
	CDH1	+			+	+	0	0	0	0	0	0	0	0	0	−	0	0	0								
	RGN	0			0	0	0	0	0	0	0	0	0	0	0	0	0	0	−								

	PDIA4				0	0	−	−	0	0	0	0	0	0	0	0	0	0	0						+		
Miscellaneous	ACTA1	0			+	0	+	+	0	0	0	0	0	0	0	−	0	0	0								

+: up-regulated

−: down-regulated

0: not modulated

The case is empty when the gene has not been studied.

Differentiated HepaRG cells from three different passages and 2-day human hepatocyte cultures from 5 donors were treated for 24 h with different concentrations of TRO. 500 ng of RNA samples from control and treated cultures were separately reverse transcribed and amplified using Quick Amplification Labeling Kit (Agilent). Then they were hybridized using 4×44 K Agilent microarrays satisfying Minimum Information About a Microarray Experiment (MIAME) requirements as previously described [82]. Normalization algorithms and background subtractions were automatically applied to each array to reduce systematic errors and to adjust effects due to technological rather than biological variations using FE and Resolver softwares. The combination of technical and biological replicates uses the error-weighted log ratio average and an estimated error method of the Rosetta Resolver system.
